# Retraction: Bone marrow mesenchymal stem cells alleviate smoke inhalation injury by regulating alveolar macrophage polarization via the CD200-CD200R pathway

**DOI:** 10.3389/fimmu.2026.1946316

**Published:** 2026-07-31

**Authors:** Frontiers Editorial Office

The journal retracts the 27 October 2025 article cited above.

Following publication, concerns were raised regarding the integrity of the images in the published figures. The authors provided raw data; however, the investigation—conducted in accordance with Frontiers’ policies—concluded that the data was not satisfactory. As a result, the findings and conclusions of the article are deemed unreliable, and the article has been retracted.

This retraction was approved by the Chief Editors of Frontiers in Immunology and the Chief Executive Editor of Frontiers. The authors were notified of the retraction and provided an opportunity to respond. This communication is on record with the publisher.

Frontiers would like to thank the users on PubPeer for bringing the published article to our attention.

